# Directly Printable Flexible Strain Sensors for Bending and Contact Feedback of Soft Actuators

**DOI:** 10.3389/frobt.2018.00002

**Published:** 2018-02-13

**Authors:** Khaled Elgeneidy, Gerhard Neumann, Michael Jackson, Niels Lohse

**Affiliations:** ^1^EPSRC Centre for Intelligent Automation, Loughborough University, Loughborough, United Kingdom; ^2^Lincoln Centre for Autonomous Systems, University of Lincoln, Lincoln, United Kingdom

**Keywords:** soft robotics, soft actuators, soft sensors, regression analysis, haptic feedback, grasping

## Abstract

This paper presents a fully printable sensorized bending actuator that can be calibrated to provide reliable bending feedback and simple contact detection. A soft bending actuator following a pleated morphology, as well as a flexible resistive strain sensor, were directly 3D printed using easily accessible FDM printer hardware with a dual-extrusion tool head. The flexible sensor was directly welded to the bending actuator’s body and systematically tested to characterize and evaluate its response under variable input pressure. A signal conditioning circuit was developed to enhance the quality of the sensory feedback, and flexible conductive threads were used for wiring. The sensorized actuator’s response was then calibrated using a vision system to convert the sensory readings to real bending angle values. The empirical relationship was derived using linear regression and validated at untrained input conditions to evaluate its accuracy. Furthermore, the sensorized actuator was tested in a constrained setup that prevents bending, to evaluate the potential of using the same sensor for simple contact detection by comparing the constrained and free-bending responses at the same input pressures. The results of this work demonstrated how a dual-extrusion FDM printing process can be tuned to directly print highly customizable flexible strain sensors that were able to provide reliable bending feedback and basic contact detection. The addition of such sensing capability to bending actuators enhances their functionality and reliability for applications such as controlled soft grasping, flexible wearables, and haptic devices.

## Introduction and Literature Review

Soft pneumatic actuators are being increasingly adopted in a wide range of applications that benefit from their inherently safe bodies and passive adaption to variations (Rus and Tolley, 2015; Hughes et al., 2016). Examples of their diverse applications include an assistive soft glove for hand rehabilitation (Polygerinos et al., 2015), a soft robotic gripper for underwater sampling of delicate species (Galloway et al., 2016), a soft mobile robot that can adapt to varying environmental conditions (Tolley et al., 2014), an autonomous soft robotic fish capable of fast body motion (Marchese et al., 2014), a soft anthropomorphic hand that can achieve complex grasp types (Deimel and Brock, 2016), and a soft manipulator inspired by the octopus arms for minimally invasive surgeries (Cianchetti et al., 2014). However, a key limitation in most soft actuators is the absence of reliable positional and force feedback, which is essential in applications that require not only a soft touch but also an accurate and controllable behavior. Hence, the idea of integrating flexible sensors to soft actuators for feedback and control purposes is being increasingly sought after in recent research. The main challenge in integrating sensors to soft actuators is the need for highly flexible sensors that do not damage the soft actuator’s body or hinder its functionality. Thus, for seamless integration of sensors into soft-bodied actuators, innovative solutions for flexible and soft sensors are required for many interesting applications, as outlined in a recent review (Amjadi et al., 2016).

A popular approach to create flexible and highly stretchable strain sensors that can be embedded into soft actuator bodies is to mix conductive additives to soft silicone rubber materials that are commonly used to create the soft actuators (Polygerinos et al., 2017). One way of achieving this is by mixing different forms of carbon additives with silicone rubbers to create stretchable soft sensors that can be easily molded (Culha et al., 2014). However, this class of sensors usually suffers from hysteresis due to the motion of conductive carbon particles within the soft matrix. This limitation has motivated later attempts to instead inject a conductive liquid metal (EGaIn) inside microchannels embedded within the silicone rubber body, to create more resilient soft and stretchable sensors that are less prone to hysteresis. The measured change in resistance from those sensors can then be related to different physical parameters such as multiaxis forces (Vogt et al., 2013) or multimodal strain and curvature (White et al., 2017). Further example applications that result from successfully embedding this type of soft sensors with soft bending actuators include position and force control of soft bending actuators using feed-forward models and a PID controller (Morrow et al., 2016), detecting the presence of a grasped object using a soft gripper (Adam Bilodeau et al., 2015), and flex and twist feedback for breaded pneumatic actuators (pneuflex) (Wall et al., 2017). Sensors with conductive liquid metal channels offer soft and highly stretchable sensors that can well bond to actuators made from similar silicone rubber materials. However, the conductive liquid metal material is expensive and injecting it to intricate channels in a soft body requires manual skills or custom printing hardware to automate the process as demonstrated more recently (Muth et al., 2014; Mohammed and Kramer, 2017).

Another approach for embedding strain sensing capability into soft actuators is the use of existing sensing components that are thin and flexible enough to not interfere with the actuator’s functionality. This property can be achieved using off-the-shelf conductive fabrics, as demonstrated by embedding pieces of conductive lycra (Electrolycra) in continuum soft arms for spatial configuration reconstruction (Cianchetti et al., 2012), as well as embedding electroconductive yarn in a soft manipulator for bending elongation feedback (Wurdemann et al., 2015). Optical fibers have also been used as macrobend stretch sensors for pose sensing in soft continuum robot arms (Sareh et al., 2015). Commercially available flex sensors are another attractive option for thin and flexible sensory elements that can be embedded within a soft actuator to change its resistance upon bending (Saggio et al., 2016). Example applications for embedding flex sensors into soft actuators include haptic identification of objects using soft gripper fingers (Homberg et al., 2015), controlling cylindrical soft actuators (Gerboni et al., 2017), and our previous work on controlling the bending of soft actuators (Elgeneidy et al., 2017). This approach offers a very simple and compact sensing solution that relies on commercially available sensors and does not require complex hardware or skills to implement. However, a known limitation with flex sensors is the significant variation between different sensor samples, as well as being prone to non-linearity and drift in their response.

The examples referenced so far involved soft actuators made mostly from silicone rubbers with low shore hardness that are inherently safe to interact with humans or delicate objects. However, they are usually fabricated manually using conventional multistage molding and curing processes (Marchese et al., 2015). Hence, a recent interest in soft robotics research is to investigate existing and new 3D printing technologies, to automate the fabrication of soft robotic components for better repeatability and accuracy as discussed in recent reviews (Truby and Lewis, 2016; Zolfagharian et al., 2016). Notable 3D printing attempts in soft robotics used advanced multi-material printers to print a functionally graded combustion-driven jumping robot (Bartlett et al., 2015), a hydraulically actuated hexapod robot (MacCurdy et al., 2015), and a soft gripper attached with a flex sensor for picking up delicate objects (Wang and Hirai, 2017). Those state-of-the-art printers allow printing of flexible materials with a range of stiffness and high precision. However, the durability and tear strength of those materials is still a limitation and removing support materials is sometimes challenging especially for intricate geometries. Other customized 3D printing technologies were proposed to print bending actuators (pouch motors) using low-cost planar printer setup (Niiyama et al., 2015), antagonistic pleated actuators using digital mask projection stereolithography (Peele et al., 2015), and stretchable elastomers using digital light processing based 3D printing (Patel et al., 2017). Those 3D printing setups can provide more affordable alternatives that are customized for specific actuator designs, but are more limited in varying the material choices and geometry of the printed actuators when compared to the first printing approach. A more recent approach is to utilize the well-established and easily accessible FDM printing technology as faster and more consistent alternative to the conventional manual fabrication, while being more affordable with better material properties compared to advanced multi-material printers. Recent work has investigated the potential of FDM printing in fabricating reliable high-force bending actuators that can be used as soft grippers (Yap et al., 2016). Another recent and relevant work involved an FDM printed soft gripper and a haptic glove with flex sensors for telemanipulation applications (Low et al., 2017).

Our work goes beyond the current state of the art by tuning a dual-extrusion FDM printing process to directly print not only highly soft bending actuators but also flexible strain sensors, using flexible and conductive materials. Our procedure results in an all-printable sensorized soft actuator that can be easily calibrated, as we demonstrate for bending and contact feedback. The sensor can be easily welded onto the actuator body since both bodies are made from the same material, offering better adhesion compared to sticking or encapsulating a commercial flex sensor to printed actuators. Hence, both the actuator and sensor are highly customizable and not restricted to off-the-shelf solutions, using easily accessible FDM printer hardware.

The paper is organized as follows: in Section “Design of Flexible Strain Sensors,” the design of the proposed flexible strain sensor is outlined, and practical guidelines for successful printing using a dual-extrusion FDM printer is detailed in Section “Dual-Extrusion FDM Printing.” Challenges with wiring and acquisition are then tackled in Section “Sensor Wiring and Acquisition” to improve the resolution and reliability of the sensor’s feedback. Subsequently, Section “Integrating to Printable Bending Actuator” discusses integration of the printed sensor to printable bending actuators to create a sensorized soft actuator module. The experimental setup for testing and characterizing the sensorized actuator using a calibrated vision system and a pneumatic control board is outlined in Section “Experimental Setup.” Moreover, the free-bending response of the sensorized actuator is characterized in Section “Characterization of the Free-Bending Response” to evaluate its consistency. The free-bending response is then calibrated in Section “Bending Angle Calibration” to derive an empirical model using linear regression. In Section “Contact Detection,” the sensorized actuator is tested in a restricted setup that prevents bending, to compare the sensor’s response to the free-bending case for contact detection purposes. Finally, the conclusions from the work are then presented with insights for future work.

## Materials and Methods

This section details the design and fabrication of the flexible strain sensor, as well as its integration to printed bending actuators.

### Design of Flexible Strain Sensors

**Figure 1:**
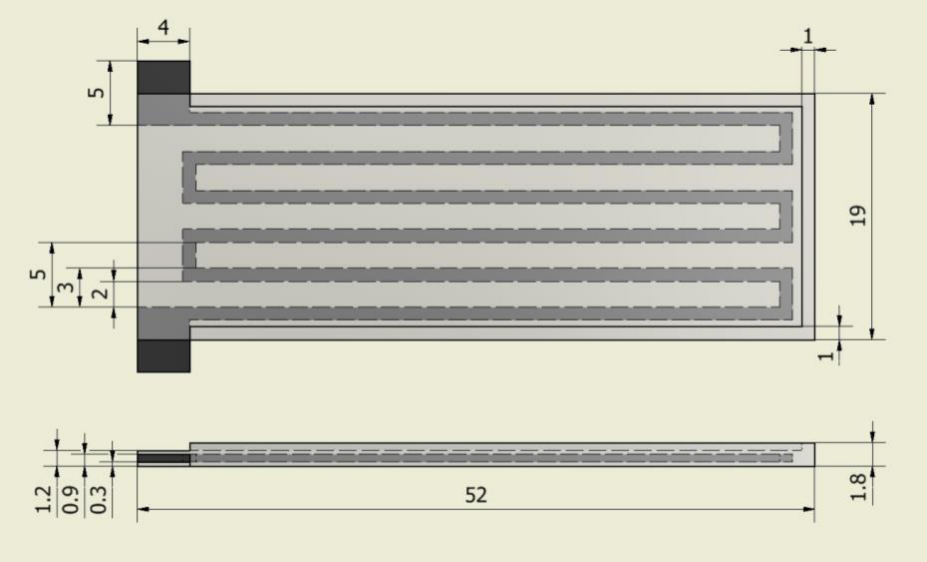
The design of the printed strain sensor (dimensions in millimeters) with flexible NinjaFlex body (gray) and embedded conductive PLA tracks (black).

The design of our flexible printed strain sensor follows that of standard strain gage sensors as shown in Figure 1. The sensor body is printed from a highly flexible material filament called NinjaFlex from NinjaTek, while the sensing tracks are printed from conductive PLA material filament from ProtoPasta. Both materials were found to bond well together when printed simultaneously. Our primary interest here is measuring the bending angle of soft actuators. Hence, the sensor should be oriented such that the channels are in line with the bending direction. Although the conductive PLA material is not very flexible, when printed with a very fine thickness it becomes flexible enough to bend, maintaining the desired flexibility of the sensor. Hence, as the flexible sensor body bends under external forces, the thin conductive tracks embedded inside will also follow the bending, causing a change in resistance. The thinnest practical track thickness is equivalent to a minimum layer thickness that the printer is able to print reliably, which was 0.3 mm for this work. However, it is recommended to make the conductive tracks out of two or three layers, so that any cavities arising during the printing of the first layer can be sealed by the following layers. This ensures functional and more resilient sensing tracks. In order to avoid excessive increase in the overall thickness of the sensor, only one layer of NinjaFlex is added on top and below the conductive tracks for sealing. Looking at the sensor’s cross section in Figure 1, it can be seen how the conductive pattern layer is encapsulated between two layers of the flexible material of 0.3 mm each. Hence, the overall thickness of the sensor is only 1.2 mm with an encapsulated sensing tracks’ thickness of only 0.6 mm. This ensures that the sensor remains highly flexible and does not limit the actuators flexibility when integrated together. Additionally, the ends of the conductive tracks are left exposed to facilitate wiring. The areas in between the conductive tracks are filled with NinjaFlex material not only to enhance the adhesion between the layers but also to create a stable all-round insulation for the conductive tracks. Finally, a raised step of 0.6 mm in height and 1 mm in width is added on top of the sensor around its circumference, in order to alleviate aligning and welding to the body of the bending actuator in the next stages.

### Dual-Extrusion FDM Printing

To enable direct 3D printing of the complete sensor in a single stage, a standard FDM printer (Lulzbut TAZ 5) fitted with a dual-extruder print head (FlexyDually^1^) was used. Having an extruder with two nozzles allows printing of the two filaments (flexible and conductive) simultaneously. Our primary target was to optimize the sensor design and print settings such that the thinnest functional sensor can be successfully printed. Increasing the cross-sectional area of the conductive tracks will ensure that they remain functional as a sensor when bent, but the flexibility of the sensor will consequently decrease as the conductive material is stiffer than the NinjaFlex material. Additionally, the dimensions of the sensing tracks must account for the printer specifications, such as nozzle diameter, so that the tracks can be printed successfully without over or under extrusion problems.

The following guidelines were followed to ensure successful and consistent printing of highly flexible and functional strain sensors.

**Figure 2:**
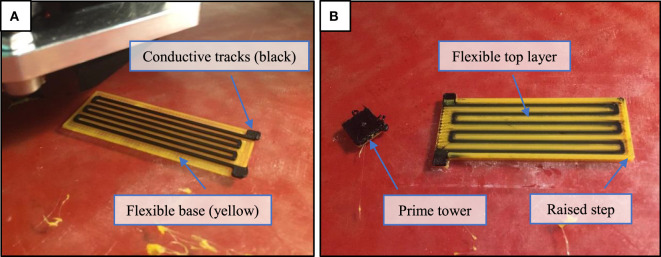
Printing the flexible strain sensor with embedded conductive tracks. **(A)** The conductive tracks printed on top of a single layer of flexible material. **(B)** The printing is complete after filling the areas between the tracks and adding the top layer with a raised step around the circumference.

- Printing orientation and direction: the sensor was printed in upright orientation in order to have full control over the geometry of the conductive tracks and ensure that they remain functional. The printing direction was set to be along the length of the conductive tracks, to minimize idle crossover between tracks, which tends to induce inconsistencies in the print.
- Design considerations: in order to ensure smooth results from the slicing software without any inconsistencies or intermitted movements, the planar dimensions of the sensor were designed to be an integral multiple of the nozzle diameter, while all vertical dimensions were chosen as an integral multiple of the printing layer height. Furthermore, the width of the conductive tracks should be at least twice as thick as the nozzle size to ensure functionality of the tracks, while limiting the vertical height to less than 0.5 mm to avoid hindering the desired flexibility. The spacing between the tracks should be at least three times larger than the nozzle diameter to ensure that no short-circuiting will occur at any point along the length of the tracks as a result of potential extra material dispensing. Maintaining a consistent spacing between the tracks is also encouraged to minimize idle tool head movements.
- Extruder switching settings: switching between nozzles is a critical source of discontinuity in the print that could negatively affect the connectivity of the conductive tracks and encourage the formation of voids between layers. The retraction settings were tuned to prevent the deposition of extra lumps that accumulate when the nozzle is idle, while ensuring that no excessive retraction happens that can delay subsequent material dispensing when the nozzle becomes active again. The retraction distance and speed for conductive PLA were set to be 10 mm/s and 4 mm at a nozzle temperature of 218°C. This was found to be the minimum nozzle temperature that allows consistent printing, as any further increase tends to cause uncontrollable dispensing of material when the nozzle is idle.
- Prime tower: the activation of this feature within the settings of the slicing software (Cura^2^) forces the printer to print a small tower with constant area (Figure 2) away from the main part when switching between materials. This option decreases the undesired dispensing from the idle nozzle since it is wiped at the tower. In addition to minimizing the dispensing delay that occurs during nozzle switching, since printing the tower first allows the flow to become consistent before printing the main part. However, this option also adds to the overall printing time.

Following the above design guidelines and print settings (available in Table S1 in the Supplementary Material), functional samples of the flexible strain sensor can be consistently printed. A significant change in resistance of around 2 kΩ in average was evident when fully bending sensor samples right after printing. This change in resistance can be directly related to the actual bending angle as explained in the following sections. As to be expected, the base resistance for the sensor and the overall change in resistance depends on the dimensions of the conductive tracks. Using the same dimensions and print settings, the variation in the base resistance value across five different samples was found to be in the range of 5% (0.5 kΩ).

### Sensor Wiring and Acquisition

**Figure 3:**
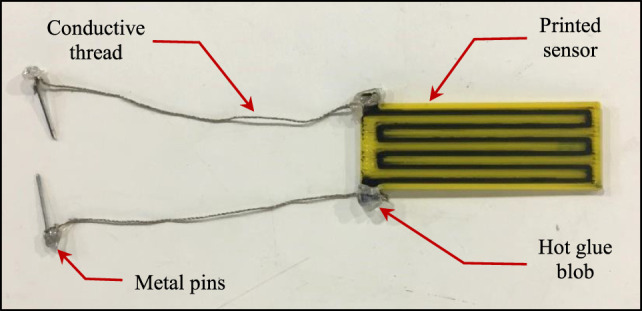
Wiring of the printed strain sensor using light conductive threads secured to the exposed terminals of the conductive tracks using hot glue.

The first challenge faced when attempting to characterize the printed strain sensor was to create a stable wiring interface. The conductive PLA material used in printing the sensing tracks cannot be soldered directly to copper or silver wires. Hence, the wires need to be clamped or tied to the sensor terminals to be able to measure the sensor’s resistance. However, when attaching the wires using metal clamps, the sensor’s measured resistance was very unstable and oscillated whenever the wires move. This effect was because the conductive tracks were printed with a very fine thickness, so attaching relatively heavier wires with rigid clamps to the track terminals introduces vibrations to the printed tracks whenever the wire moves, which disturb the measured readings. Additionally, the metal clamps are much harder than the flexible sensor, so they can easily damage the sensor terminals after repeated use. In order to resolve this problem, a different wiring technique is proposed that uses low-cost conductive threads^3^ (commonly used for wearable applications) as wires. The thread is simply wound around the sensor terminals and hot glue is applied to fix the conductive thread in place, see Figure 3. The other end of the conductive thread is again wound around a metal pin and fixed using hot glue, to facilitate connecting the sensor to circuit boards (Figure 3). This results in a light and flexible wire that can be fixed securely to the sensor without damaging the terminals or influencing the sensor reading by the wires’ weight. Using this alternative wiring approach, the measured sensor resistance no longer fluctuated significantly due to the movement of the wires and stable readings could be recorded.

**Figure 4:**
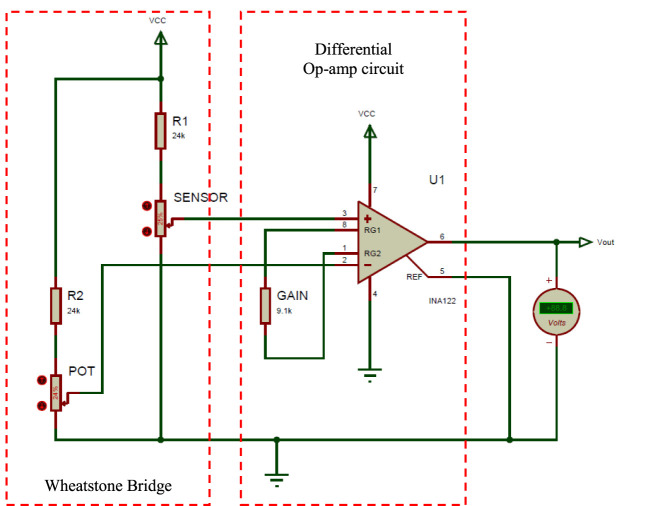
Schematic diagram of the signal conditioning circuit that converts and amplifies the change in resistance of the printed sensor due to bending into voltage.

A limitation of our approach that becomes more evident as it is used for a longer duration was the drift in the printed sensor’s readings. This limitation is often associated with resistive-based sensors, due to heating of the sensor as current passes through its conductive tracks during operation. The drift due to heating would continue to increase the longer the sensor is used, making it difficult to accurately characterize the sensor response. Possible solutions for this drift problem include cooling the sensor to a fixed temperature, or using an additional dummy sensor as part of a Wheatstone bridge circuit to negate the change in measurement due to heating. However, those are not practical solutions for the envisioned applications as we require the sensor to be directly attached to an actuator in a confined space. Thus, the drift effect was instead minimized using a signal conditioning circuit that reduces the current passing through the sensor and adds a bias function to reset the output for longer operations. Figure 4 shows a schematic diagram for the signal conditioning circuit that was designed to: (1) convert the sensor’s change in resistance into voltage, (2) amplify the output voltage across the 0–5 V range, (3) minimize the drift in readings due to heating, and (4) add a bias function. The circuit comprises a Wheatstone bridge circuit that is balanced at the sensor’s base resistance value, and thus outputs a voltage corresponding to the change in the sensor’s resistance when bent. This bridge is followed by an instrumentation amplifier IC (INA122, Texas Instruments) that amplifies only the change in resistance due to the sensor’s bending for a better measurement resolution. Adding the sensor as part of the bridge reduced the current passing through the sensor’s conductive tracks, which again reduces the heating and the resulting drift.

The value of R1 was set to be within the range of resistance values for the printed sensor, which was 24 kΩ. The bridge is initially balanced by setting the same resistance value for resistors R2 and using a potentiometer to 0 the final voltage output due to any errors in the resistance values when the sensor is flat. The amplification gain of the circuit is set using the resistance (GAIN). The output voltage from the signal conditioning circuit was simulated against the change in the printed sensor resistance for gain values of 34.4, 26.9, and 20, which were set using standard resistance values of 6.8, 9.1, and 13.3 kΩ, respectively. It was found that a gain of 26.9 (*R*_gain_ = 9.1 kΩ) results in the most linear voltage response across the range of sensor resistance values and hence, this value was used. The corresponding voltage output from the circuit in this case was ranged from 0.4 to 4.8 V, which effectively utilizes the 5 V range for enhanced resolution. This output voltage is then fed to an analog pin of the Arduino board to convert it to a digital reading (from 0 to 1,024).

### Integrating to Printable Bending Actuator

**Figure 5:**
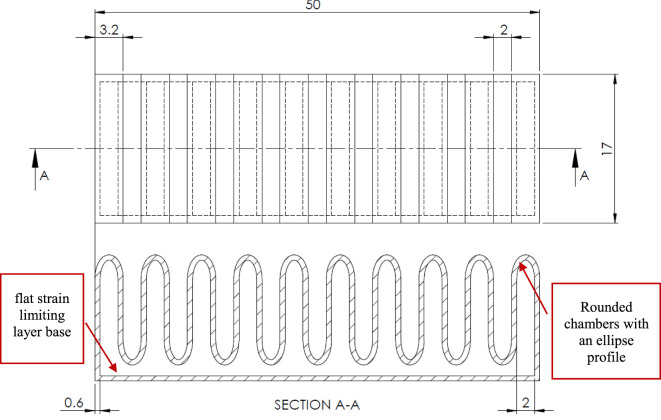
Top and cross-sectional front views for the design of the printable bending actuator following a pleated morphology (dimensions in millimeters).

**Figure 6:**
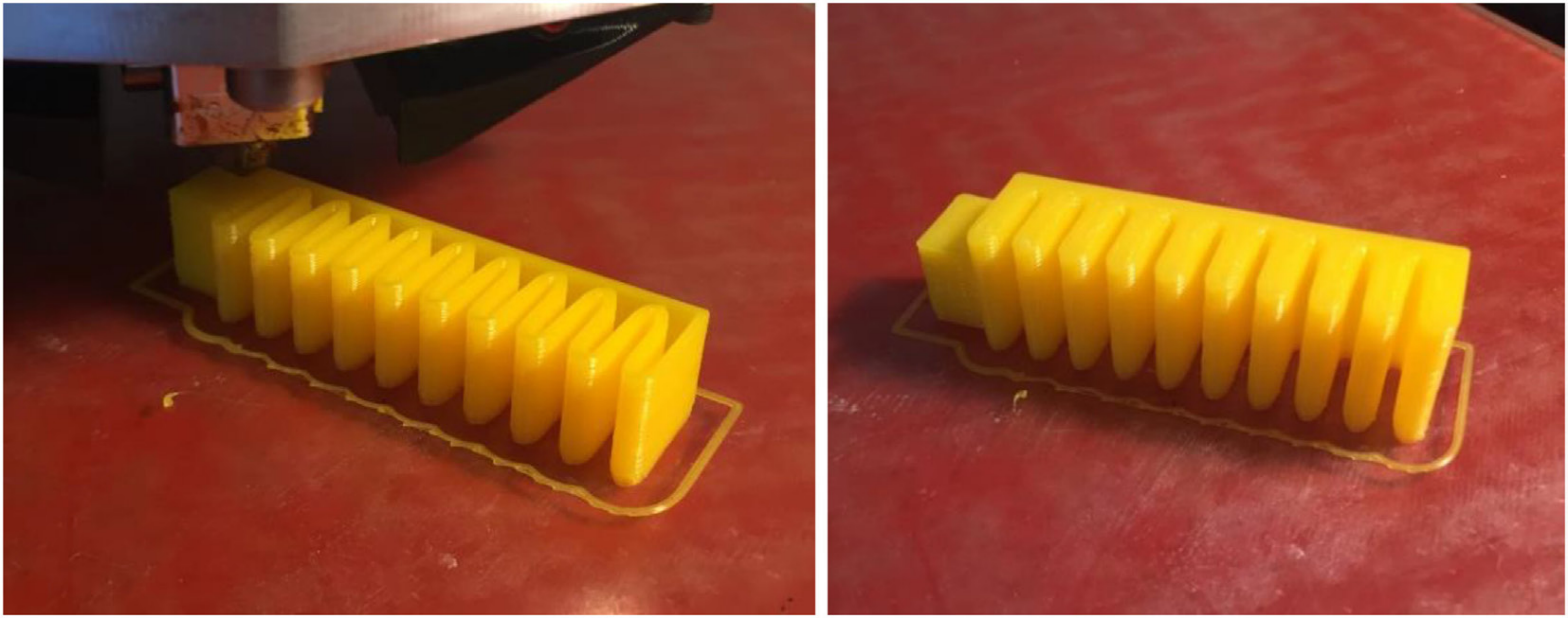
Printing a bending actuator sample in a sideway orientation, before and after sealing the top layers.

An application for the printed strain sensor considered in this paper is the generation of bending and contact feedback for soft bending actuators. This section presents a directly printable bending actuator that is soft and flexible, using the common FDM printing technology. The design of the printable bending actuator is based on the same concept of conventional soft bending actuators, in which chambers are pressurized to expand while the base is constrained to generate a bending motion. Considering the fact that the flexible NinjaFlex material that we used in printing is not as flexible as commonly used silicone rubber materials, we adopted a pleated morphology with separated chambers (Figure 5) to minimize the material strain (Marchese et al., 2015). Additionally, the actuator chambers were rounded with an ellipse profile to further improve the actuator’s flexibility. Similar work has been recently published that successfully demonstrated the reliability of FDM printing in producing high-force bending actuators (Yap et al., 2016). Yet, our bending actuator was successfully printed with a thinner shell thickness of 0.6 mm and bottom/top layer thickness of 1.2 mm, with the addition of the flexible strain sensor for bending and contact feedback. The actuator was printed in sideway orientation to minimize the bridging distance, during which the printer nozzle is dispending a new material layer without a supporting layer beneath as shown in Figure 6. A pneumatic fitting is then tapped to the actuator’s inlet to simplify the connection to the pneumatic supply. The tuned printing parameters that were used to print the actuator are provided in Table S2 in the Supplementary Material.

**Figure 7:**
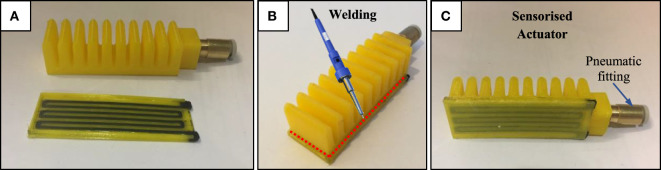
Welding the printed bending actuator and strain sensor together to create a sensorized actuator. **(A)** Individual actuator and sensor after printing, **(B)** soldering iron used to weld along the raised step of the sensor, **(C)** sensorized actuator after welding.

To be able to test the printed strain sensor systematically under consistent bending conditions and evaluate its application for providing bending and contact feedback, the sensor was welded to the printed actuator. Hence, each of the actuator and sensor are separately printed using individual printing configurations without the need for any additional postprocessing to ensure functionality. The soft actuator body is printed sideways to minimize bridging and ensure air tightness, while the sensor is printed upright to have better control over the geometry of the conductive tracks and ensure that they are thin enough to maintain flexibility. Merging the two parts together is then achieved by simply welding along the raised edge of the sensor using a soldering iron, as shown in Figure 7, to locally melt the NinjaFlex material and bond them together. This additional step, although manual, is simple and quick to achieve, while allowing each part to be printed following their optimized printing settings. We also tried to directly print the actuator with the sensor; however, the output was not consistent, as either the actuator or the sensor was not functioning properly. Hence, this printing and welding approach was preferred, in order to ensure consistently air-tight actuators and functional strain sensors.

### Experimental Setup

**Figure 8:**
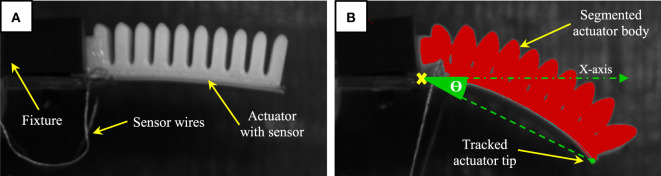
**(A)** Printed soft actuator with integrated sensor fixed in place for testing. **(B)** A processed image frame sample during actuation showing the segmented soft actuator body and tracked tip.

To evaluate the sensor’s response, we systematically tested and characterized our sensorized soft actuator using controlled input conditions. The sensorized actuator is secured using a 3D printed fixture to a frame and connected to a pneumatic supply of controlled pressure using a pneumatic control board.^4^ The wiring from the printed strain sensor is connected to the designed accusation circuit for preprocessing, before reaching the analog input of the Arduino board on the pneumatic control board. The internal pressure reaching the actuator is also measured using onboard pressure sensor (Honeywell-ASDXAVX100PGAA5) and fed to the Arduino board. At the other end of the mounting frame, a high-speed camera (MAKO G-223) is secured and set to capture image frames at 130 fps. The camera was also calibrated for taking measurements in real-world coordinates with a mean error of 0.01 mm at a focal length of 17.5 mm. We developed an image processing program using the Halcon library^5^ to analyze the captured image frames (Figure 8) to (a) automatically identify the actuator body, (b) record the coordinates of the detected actuator tip, and (c) calculate the bending angle at each captured frame. The bending angle is measured between the horizontal axis aligned with the bottom of the actuator in its original flat position, and the line connecting a fixed reference point at the base of the actuator to the detected actuator tip as illustrated in Figure 8. The procedure for testing our printed soft actuator with integrated strain sensor is as follows:

- The desired input pressure supply to the actuator is set.
- The actuator is fixed to the setup and actuated repeatedly while recording the image frames and sensory feedback.
- The output voltage from the pressure and bending sensors are converted to digital values *via* the analog inputs of the Arduino board and recorded as a time series.
- The captured image frames are stored on the PC and processed using the image processing program to track the tip trajectory and calculate the corresponding bending angle value for each image frame.
- The bending angle values are synchronized with the processed readings from the strain sensor and onboard pressure sensor.

## Results

The sensorized actuator has been systematically tested to characterize its free-bending response and calibrate the sensor’s response against measurements made by the vision system. This is followed by testing the sensorized actuator again in contact state and comparing that to the free-bending response, in order to evaluate the potential of achieving simple contact detection.

### Characterization of the Free-Bending Response

**Figure 9:**
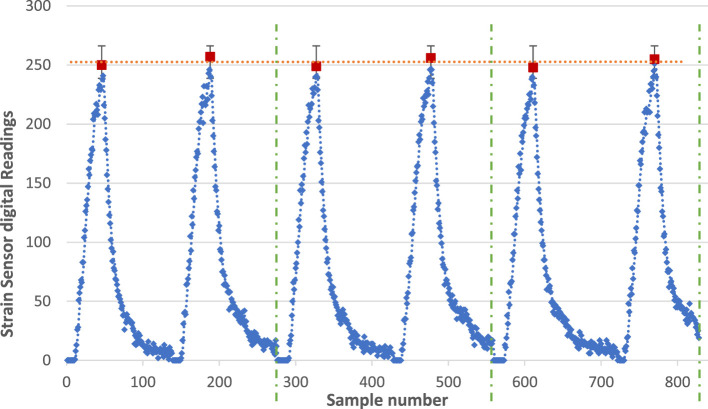
Sample voltage output (after analog to digital conversion) from the conditioning circuit when repeatedly bending the sensor.

The consistency of the readings from the printed strain sensor is evaluated while welded to the printed actuator, so that it bends consistently with the actuator when pneumatically actuated. The resulting readings from the sensor are recorded *via* the analog input of the Arduino board and converted to digital values between 0 and 1,024 (corresponding to 0–5 V). Figure 9 shows the response of the printed strain sensor when the actuator is tested six times at a fixed pressure input of 18 psi. Six clear cycles can be seen in the graph, each corresponds to the sensor’s response during the actuation and retraction phases. The average final value from the six cycles was 252 with an SD of 3.59 and a maximum error of 7 units (corresponding to only 0.034 V). This test illustrates the consistency of the processed sensor’s response for fixed input conditions.

**Figure 10:**
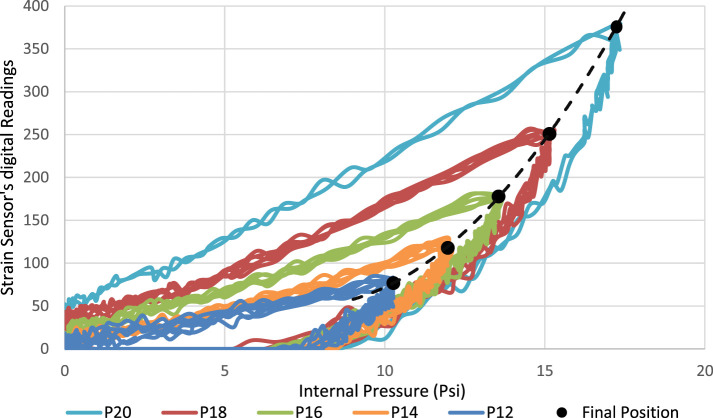
Printed strain sensor’s response when welded to a printed bending actuator against the internal pressure at variable input pressure values.

Furthermore, in order to evaluate the consistency of the sensor’s response across variable input pressure, the sensorized soft actuator was tested three times at pressure inputs from 12 to 20 psi at 2 psi increments. In Figure 10, the readings (after analog to digital conversion) from the strain sensor were plotted against the actuator’s internal pressure measured using the onboard pressure sensor. Five consistent cycles can be seen in the graph, each representing the response for a given pressure input. The response is increasing (actuation stage) following a consistent parabolic response for each input pressure value, then starts to fall back (retraction stage) once the input pressure is switched off. The gradient of the response increases with increased input pressure, while the retraction phase for all input pressures almost follows the same curve since retraction is mainly governed by the elasticity of the actuator when no pressure is supplied. The clear difference between the actuation and retraction paths means that the sensor’s response exhibits hysteresis, as it is the case with similar resistive-based sensors. Plotting a curve through the final position of the sensorized actuator for each cycle yields a polynomial function that describes the relation between the input pressure and maximum bending. This experiment confirms that a consistent response from the sensorized actuator can be identified for each input pressure, which can be described by simple polynomial functions.

### Bending Angle Calibration

**Figure 11:**
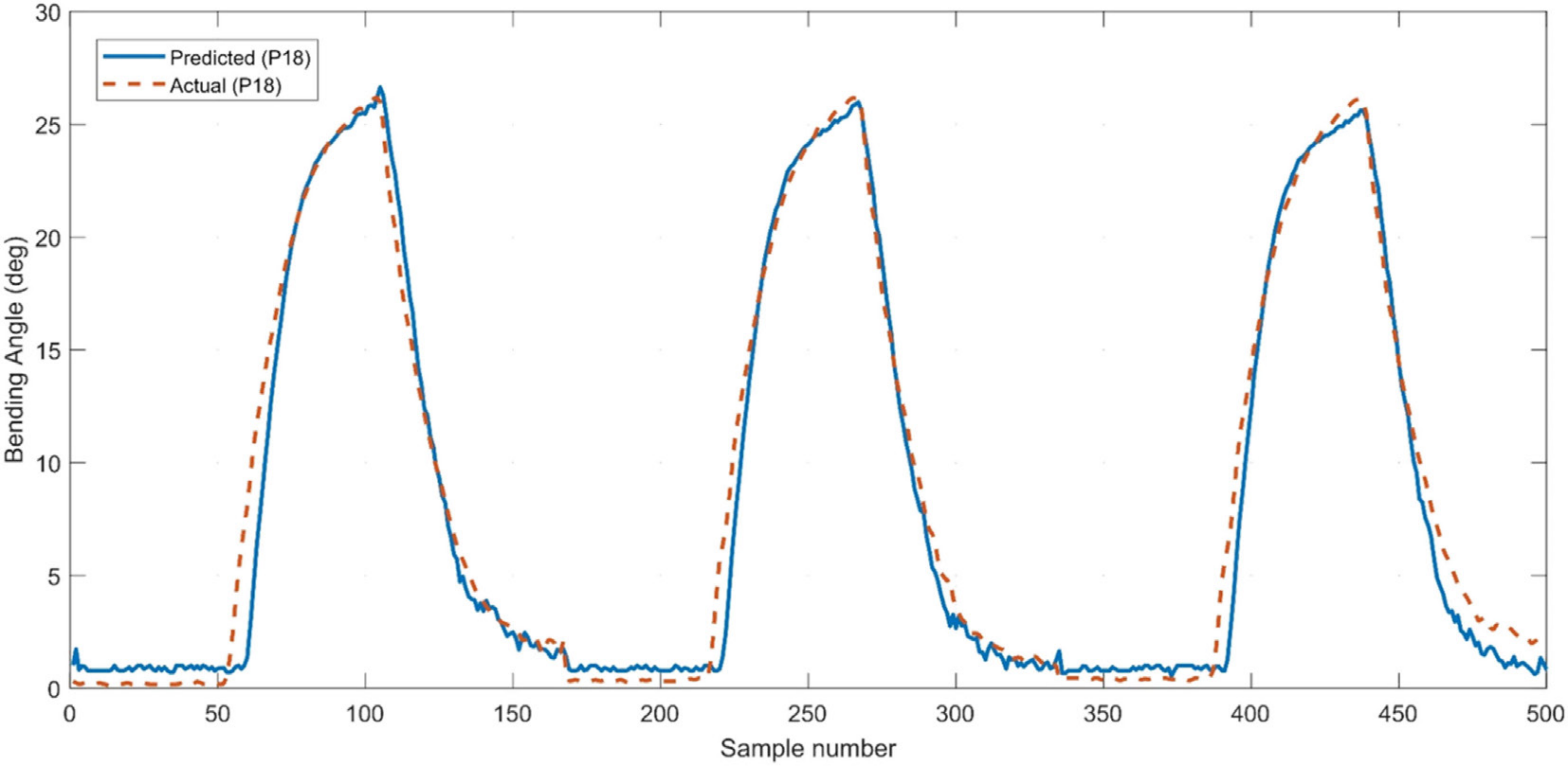
Evaluation of the model accuracy in reproducing the training dataset acquired by testing the sensorized actuator at an input pressure of 18 psi.

In the next stage, we calibrated the voltage measurement from the sensorized printed actuator, based on real measurements of the bending angle acquired using the calibrated vision system. Simple linear regression implemented in Matlab was used to estimate the polynomial relationship between the generated sensory readings and the corresponding bending angle. Two sets of experiments were conducted to generate the training and validation datasets required for deriving the empirical relationship. In the first experiment, the sensorized actuator was tested three times at a fixed input pressure of 18 psi, while recording the resulting sensory readings and the corresponding bending angle values. A total of 501 samples were collected at a sampling rate of 5 ms. Each sample is an array containing a voltage reading from each of the integrated printed sensor and the onboard pressure sensor, as well as the corresponding synchronized measurement of the actual bending angle from the vision system. The internal pressure is added as a variable in the model, because varying the input pressure changes the slope of the bending response as witnessed in the previous tests. Eq. 1 shows the normalized coefficients of the resulting second-order polynomial function, with an *R*^2^ value of 0.98 and RMSE of only 0.872°. The input variables to the model are the readings from the strain sensor (*S*) and internal pressure (*P*). Figure 11 illustrates the prediction accuracy of the model in comparison to the target bending angle values measured by the vision system.

Equation 1: derived polynomial function from the regression analysis

(1) $$\theta =10.77+3.63S+8.386P+1.055S^{2}-3.532SP\;$$

where θ: bending angle, *S*: strain sensor’s reading, and *P*: internal pressure.

**Figure 12:**
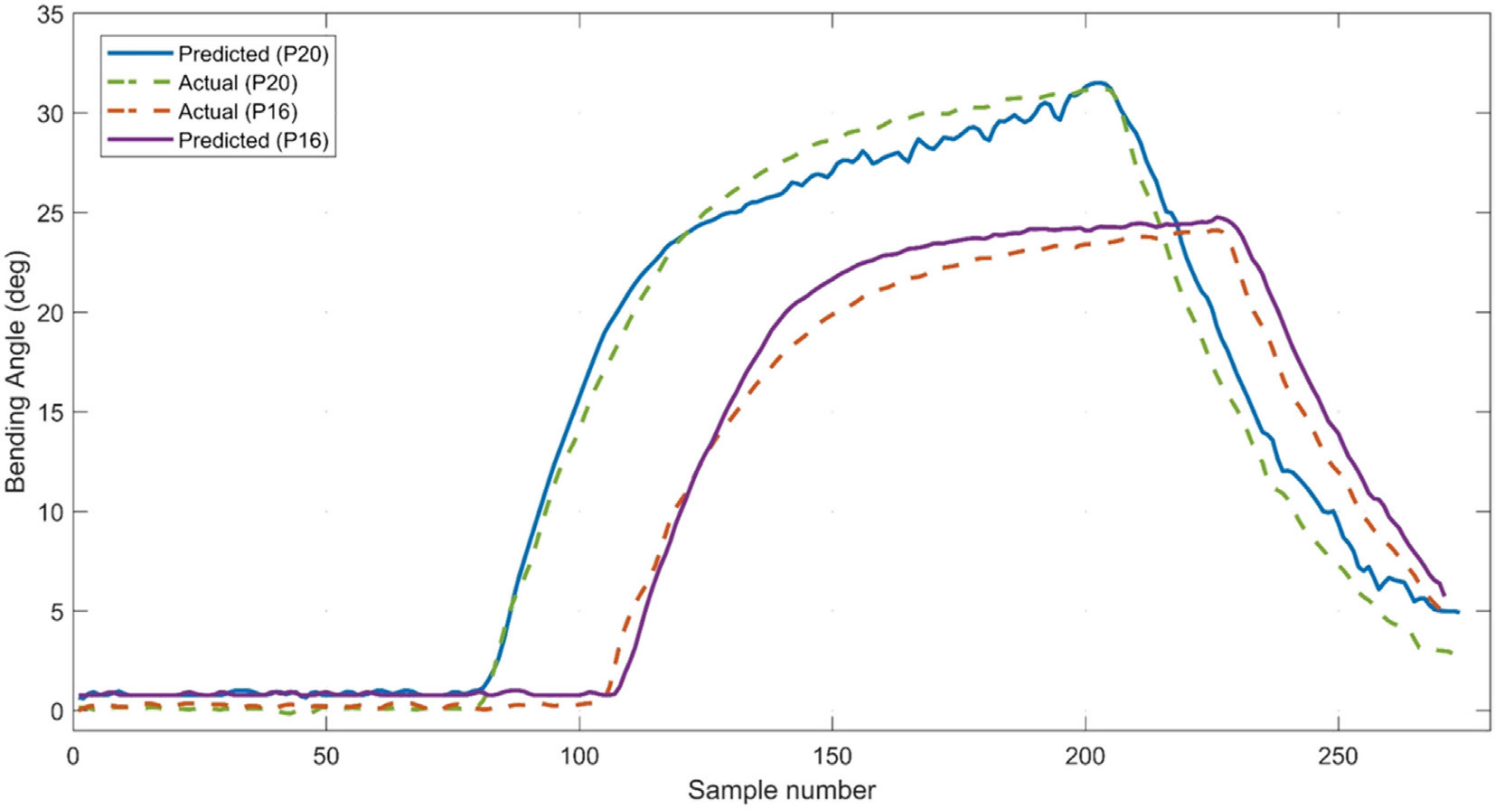
Comparing the predicted bending values to the actual values when testing the derived model on untrained input pressures of 16 and 20 psi.

We validated the derived model using new experimental data that was not used in the training process. To do so, a second experiment was conducted in which the sensorized bending actuator was tested at untrained input pressures of 16 and 20 psi. The new generated dataset of 550 samples in total was fed to the model to compare the predicted bending angle to the actual bending angle measured using the vision system. The actual and predicted bending angles for each test are plotted in Figure 12 with an RMSE of 1.29°. It can be observed that the predicted response closely follows that of the actual response, which confirms that the training did not overfit the model to the input conditions of the training data. The result also highlights the benefit of including the internal pressure variable in the model, in order to generate accurate bending angle estimations at variable input pressures. However, the accuracy of the model is expected to be influenced by any variations in printing the sensorized actuator. Yet, as we demonstrated, the model can always be tuned using a relatively small amount of new training data to achieve acceptable results. Alternative methods to linear regressions such as feed-forward neural networks could also result in even more accurate predictions. However, our regression analysis still provided accurate results and has the benefit of generating simple models that can be easily utilized as part of a closed-loop controller for real-time control.

### Contact Detection

**Figure 13:**
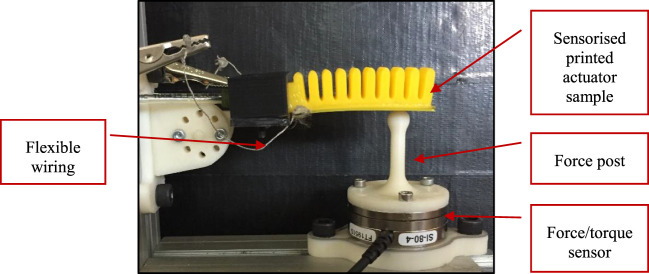
Setup for measuring the contact forces generated by the sensorized actuator during partially restricted actuation.

Another feature of our printable strain sensors is the ability to detect contact by comparing the sensor’s response upon making contact to that of the known free-bending response at the same input pressure. In order to evaluate this capability, the same experimental setup previously outlined was used with the addition of a force/torque sensor (Schunk mini40) fitted with a 3D printed force post on top (Figure 13). The assembly is placed beneath the actuator’s tip to restrict the actuator’s tip bending and measure the forces generated upon contact, while recording the corresponding readings from the strain sensor. The measured forces largely depend on the location of the force post and will not represent the maximum force capability of the sensorized actuator, since it is not completely restricted. However, our aim was to study the embedded sensor’s response when making contact with an object in a typical interaction scenario and not to quantify its maximum force generation.

**Figure 14:**
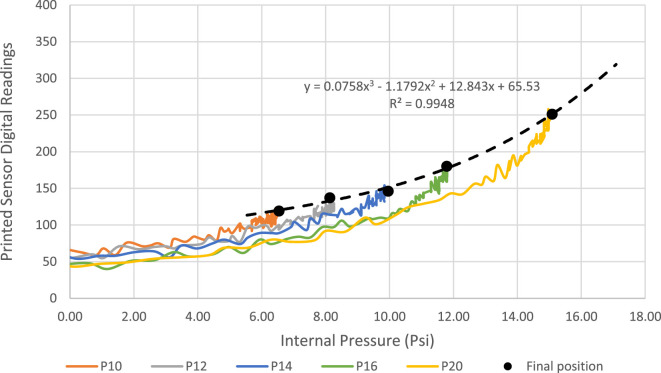
Integrated strain sensor measurements (after signal acquisition) at different input pressures during contact tests.

The sensorized soft actuator was actuated twice at input pressures ranging from 12 to 20 psi with 2 psi increments. Figure 14 shows the resulting voltage measurements from the integrated flexible strain sensor during those contact force tests. It is observed that the readings from the sensor still increased even though the actuator has been mostly in contact with the force post. We believe that the primary reason for this effect is because the sensor is pushed against the force post causing compressive forces that brings the printed layers of the sensing tracks closer together. As a result, the overall resistivity of the sensor increases, contributing to the observed increase in the measured output voltage. Additionally, the sensor still exhibits slight bending across its body since it is not completely restrained from the top, which also contributes to the witnessed increase in output voltage. This would normally be the case for the sensorized actuator’s intended applications, as the interaction with targets would be mostly at the tip without completely restricting the bending of the entire body.

**Figure 15:**
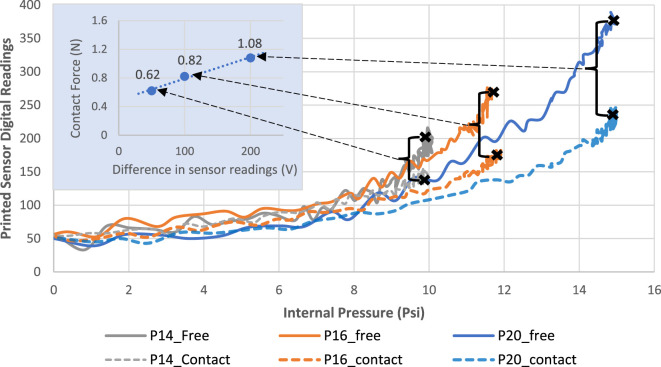
Comparing the free-bending response of the embedded sensor to the contact response at three different pressure inputs.

To highlight the potential for using the integrated strain sensor for contact detection, its response when the actuator is in a contact state is compared to the free-bending response at three different input pressure values. Figure 15 shows the noticeable difference in response for the contact and free states when tested at pressures of 14, 16, and 20 psi, which is more significant at higher input pressures. Consequently, even though the readings do continue to increase upon making contact, the readings acquired for the free-bending scenario at the same operating conditions increased with a higher rate as highlighted in Figure 15. It is also observed that increasing the input pressure when the actuator is constrained does not cause a significant increase in the slope of the response as previously witnessed with the free-bending response (Figure 10). Hence, we can distinguish when a contact is made by comparing the real-time sensory response to that of the known free-bending response at the same input pressure. This approach provides a simple method for detecting contact during typical grasping applications without the need for additional sensors. Furthermore, Figure 15 also shows the difference in the sensor readings between the free and contact responses, versus the corresponding resultant contact force measured by the force/torque sensor. The graph indicates a directly proportional relationship between the difference in the sensor readings and the measured resultant forces. This relation is largely dependent on several other factors including the contact location and the nature of the target object, which were fixed for this test to facilitate the comparison. Nevertheless, this experiment showed that the difference in the sensor’s readings between the free and contact responses can be used to infer the strength of the contact forces. Acquiring a precise value for the contact forces will require more sophisticated tactile sensors; yet, our simple printed sensor can still provide useful insights for applications where the exact value of the contact forces is not necessary.

## Discussion

In summary, the outcomes of this work demonstrated how the well-established FDM printing process can reliably produce highly flexible sensorized soft actuators for accurate bending feedback and simple contact detection. The soft bending actuator followed a pleated morphology and was successfully printed using an FDM printer, as demonstrated by recent similar work. The work goes further beyond the state of the art in this direction by designing, printing, and calibrating flexible strain sensors, which can be easily welded to the printed actuator’s body. Our approach yields an all-printable sensorized soft actuator that can be easily customized based on the application needs. The flexible strain sensors were printed using a tuned dual-extrusion FDM process, which allowed printing of flexible and conductive filaments simultaneously. The main advantage of our approach compared to using commercially available sensors is the fact that low-cost sensors can be easily customized and quickly fabricated using accessible FDM printer hardware. However, the main limitations of the sensor were the presence of drift due to current heating that becomes more evident after prolonged use and the presence of hysteresis when comparing the actuation and retraction responses. Yet, those limitations are commonly evident with resistive-based sensors. The drift effect was however minimized using the developed signal conditioning circuit and the addition of the bias option to reset the output when needed. Moreover, systematic experimental analysis under variable input pressures showed that the sensorized bending actuators exhibit a consistent free-bending response. A calibration model was derived using regression analysis to convert the sensor readings from the embedded strain sensors and the onboard pressure sensor, to bending angle values measured using a calibrated vision system. The model accurately represented the bending angle response with an *R*^2^ value of 0.98 and RMSE of only 0.872° and was successfully validated using untrained data at different input pressures with an RMSE of 1.29°. Finally, the sensorized actuator was tested in a partially constrained setup in the presence of a force post mounted on top of a force/torque sensor to measure the generated contact forces at the actuator’s tip, while recording the corresponding strain sensor’s response. This partially constrained configuration is expected to be witnessed during typical applications, such as grasping, in which the sensorized actuator would make contact with the target object at its tip, but can still exhibit some limited bending since the rest of its body is not completely restrained. The results showed a clear difference in the sensor’s response when compared to the free-bending case at the same input pressure. This highlighted the potential of using the sensor for simple contact detection based on prior knowledge of the free-bending response. Additionally, we demonstrated that the difference in the sensory measurements between the constrained and free-bending responses can be utilized to make simple inferences regarding the strength of the contact forces, which is a useful feature when grasping or interacting with delicate objects.

Future work will involve creating a complete printable soft gripper with integrated sensing capability that is based on our calibrated sensorized actuators. The presence of the bending and contact feedback will enhance the capabilities of such a soft gripper by detecting contact with target objects, estimating their size, and making simple inferences regarding the grasp quality. Such highly customizable soft grippers will be inexpensive and fast to fabricate, which is useful for applications requiring a delicate and more controllable grasping.

## Author Contributions

KE: conceived the research work and wrote the manuscript with critical input and review from NL and GN. NL: supervised and guided the research, providing helpful feedback during the implementation of the work and preparation of the manuscript. GN: reviewed the manuscript, providing useful comments and feedback. MJ: second supervisor of the research project.

## Conflict of Interest Statement

The authors declare that the research was conducted in the absence of any commercial or financial relationships that could be construed as a potential conflict of interest.

## Footnotes

^1^https://www.lulzbot.com/store/tool-heads/lulzbot-taz-flexydually-tool-head-v2.

^2^https://www.lulzbot.com/cura.

^3^https://www.proto-pic.co.uk/conductive-thread-bobbin-60ft.html.

^4^https://softroboticstoolkit.com/book/control-board.

^5^http://www.mvtec.com/products/halcon/.

## References

[B1] Adam Bilodeau R. White E. L. Kramer R. K. (2015). “Monolithic fabrication of sensors and actuators in a soft robotic gripper,” in IEEE International Conference on Intelligent Robots and Systems (Hamburg, Germany), 2324–2329.

[B2] Amjadi M. Kyung K.-U. Park I. Sitti M. (2016). Stretchable, skin-mountable, and wearable strain sensors and their potential applications: a review. Adv. Funct. Mater. 26, 1678–1698. 10.1002/adfm.201504755

[B3] Bartlett N. W. Tolley M. T. Overvelde J. T. B. Weaver J. C. Mosadegh B. Bertoldi K. (2015). A 3D-printed, functionally graded soft robot powered by combustion. Science 349, 161–165. 10.1126/science.aab0129 26160940

[B4] Cianchetti M. Ranzani T. Gerboni G. Nanayakkara T. Althoefer K. Dasgupta P. (2014). Soft robotics technologies to address shortcomings in today’s minimally invasive surgery: the STIFF-FLOP approach. Soft Robot. 1, 122–131. 10.1089/soro.2014.0001

[B5] Cianchetti M. Renda F. Licofonte A. Laschi C. (2012). “Sensorization of continuum soft robots for reconstructing their spatial configuration,” in Proceedings of the IEEE RAS and EMBS International Conference on Biomedical Robotics and Biomechatronics (Roma, Italy), 634–639.

[B6] Culha U. Nurzaman S. G. Clemens F. Iida F. (2014). SVAS3: strain vector aided sensorization of soft structures. Sensors 14, 12748–12770. 10.3390/s140712748 25036332 PMC4168483

[B7] Deimel R. Brock O. (2016). A novel type of compliant and underactuated robotic hand for dexterous grasping. Int. J. Robot. Res. 35, 161–185. 10.1177/0278364915592961

[B8] Elgeneidy K. Lohse N. Jackson M. (2017). Bending angle prediction and control of soft pneumatic actuators with embedded flex sensors – a data-driven approach. Mechatronics. 10.1016/j.mechatronics.2017.10.005

[B9] Galloway K. C. Becker K. P. Phillips B. Kirby J. Licht S. Tchernov D. (2016). Soft robotic grippers for biological sampling on deep reefs. Soft Robot. 3, 23–33. 10.1089/soro.2015.0019 27625917 PMC4997628

[B10] Gerboni G. Diodato A. Ciuti G. Cianchetti M. Menciassi A. (2017). Feedback control of soft robot actuators via commercial flex bend sensors. IEEE ASME Trans. Mech. 22, 1–1. 10.1109/TMECH.2017.2699677

[B11] Homberg B. S. Katzschmann R. K. Dogar M. R. Rus D. (2015). “Haptic identification of objects using a modular soft robotic gripper,” in IEEE International Conference on Intelligent Robots and Systems (Hamburg, Germany), 1698–1705.

[B12] Hughes J. Culha U. Giardina F. Günther F. Rosendo A. (2016). Soft manipulators and grippers: a review. Front. Robot. AI 3:69 10.3389/frobt.2016.00069

[B13] Low J. H. Lee W. W. Khin P. M. Thakor N. V. Kukreja S. L. Ren H. L. (2017). Hybrid tele-manipulation system using a sensorized 3D-printed soft robotic gripper and a soft fabric-based haptic glove. IEEE Robot. Autom. Lett. 2, 880–887. 10.1109/LRA.2017.2655559

[B14] MacCurdy R. Katzschmann R. Kim Y. Rus D. (2015). Printable hydraulics: a method for fabricating robots by 3D co-printing solids and liquids. arXiv Prepr. arXiv1512.03744.

[B15] Marchese A. D. Katzschmann R. K. Rus D. (2015). A recipe for soft fluidic elastomer robots. Soft Robot. 2, 7–25. 10.1089/soro.2014.0022 27625913 PMC4997626

[B16] Marchese A. D. Onal C. D. Rus D. (2014). Autonomous soft robotic fish capable of escape maneuvers using fluidic elastomer actuators. Soft Robot. 1, 75–87. 10.1089/soro.2013.0009 27625912 PMC4997624

[B17] Mohammed M. G. Kramer R. (2017). All-printed flexible and stretchable electronics. Adv. Mater. 29. 10.1002/adma.201604965 28247998

[B18] Morrow J. Shin H. S. Phillips-Grafflin C. Jang S. H. Torrey J. Larkins R. (2016). “Improving soft pneumatic actuator fingers through integration of soft sensors, position and force control, and rigid fingernails,” in Proceedings - IEEE International Conference on Robotics and Automation (Stockholm, Sweden), 5024–5031.

[B19] Muth J. T. Vogt D. M. Truby R. L. Mengüç Y. Kolesky D. B. Wood R. J. (2014). Embedded 3D printing of strain sensors within highly stretchable elastomers. Adv. Mater. 26, 6307–6312. 10.1002/adma.201400334 24934143

[B20] Niiyama R. Sun X. Sung C. An B. Rus D. Kim S. (2015). Pouch motors: printable soft actuators integrated with computational design. Soft Robot. 2, 59–70. 10.1089/soro.2014.0023

[B21] Patel D. K. Sakhaei A. H. Layani M. Zhang B. Ge Q. Magdassi S. (2017). Highly stretchable and UV curable elastomers for digital light processing based 3D printing. Adv. Mater. 29. 10.1002/adma.201606000 28169466

[B22] Peele B. N. Wallin T. J. Zhao H. Shepherd R. F. (2015). 3D printing antagonistic systems of artificial muscle using projection stereolithography. Bioinspir. Biomim. 10, 55003. 10.1088/1748-3190/10/5/055003 26353071

[B23] Polygerinos P. Correll N. Morin S. A. Mosadegh B. Onal C. D. Petersen K. (2017). Soft robotics: review of fluid-driven intrinsically soft devices; manufacturing, sensing, control, and applications in human-robot interaction. Adv. Eng. Mater. 1–22. 10.1002/adem.201700016

[B24] Polygerinos P. Wang Z. Galloway K. C. Wood R. J. Walsh C. J. (2015). Soft robotic glove for combined assistance and at-home rehabilitation. Rob. Auton. Syst. 73, 135–143. 10.1016/j.robot.2014.08.014

[B25] Rus D. Tolley M. T. (2015). Design, fabrication and control of soft robots. Nature 521, 467–475. 10.1038/nature14543 26017446

[B26] Saggio G. Riillo F. Sbernini L. Quitadamo L. R. (2016). Resistive flex sensors: a survey. Smart Mater. Struct. 25, 13001 10.1088/0964-1726/25/1/013001

[B27] Sareh S. Noh Y. Li M. Ranzani T. Liu H. Althoefer K. (2015). Macrobend optical sensing for pose measurement in soft robot arms. Smart Mater. Struct. 24, 125024 10.1088/0964-1726/24/12/125024

[B28] Tolley M. T. Shepherd R. F. Mosadegh B. Galloway K. C. Wehner M. Karpelson M. (2014). A resilient, untethered soft robot. Soft Robot. 1, 213–223. 10.1089/soro.2014.0008

[B29] Truby R. L. Lewis J. A. (2016). Printing soft matter in three dimensions. Nature 540, 371–378. 10.1038/nature21003 27974748

[B30] Vogt D. M. Park Y.-L. Wood R. J. (2013). Design and characterization of a soft multi-axis force sensor using embedded microfluidic channels. IEEE Sens. J. 13, 4056–4064. 10.1109/JSEN.2013.2272320

[B31] Wall V. Zoller G. Brock O. (2017). “A method for sensorizing soft actuators and its application to the RBO hand 2,” in Proceedings - IEEE International Conference on Robotics and Automation (Singapore), 4965–4970.

[B32] Wang Z. Hirai S. (2017). “A 3D printed soft gripper integrated with curvature sensor for studying soft grasping,” in SII 2016 – 2016 IEEE/SICE Int. Symp. Syst. Integr, 629–633.

[B33] White E. L. Case J. C. Kramer R. K. (2017). Multi-mode strain and curvature sensors for soft robotic applications. Sens. Actuators A Phys. 253, 188–197. 10.1016/j.sna.2016.11.031

[B34] Wurdemann H. A. Sareh S. Shafti A. Noh Y. Faragasso A. Chathuranga D. S. (2015). “Embedded electro-conductive yarn for shape sensing of soft robotic manipulators,” in Proc. Annu. Int. Conf. IEEE Eng. Med. Biol. Soc. EMBS, Vol. 2015, 8026–8029. 10.1109/EMBC.2015.7320255 26738155

[B35] Yap H. K. Ng H. Y. Yeow C.-H. (2016). High-force soft printable pneumatics for soft robotic applications. Soft Robot. 3, 144–158. 10.1089/soro.2016.0030

[B36] Zolfagharian A. Kouzani A. Z. Khoo S. Y. Moghadam A. A. A. Gibson I. Kaynak A. (2016). Evolution of 3D printed soft actuators. Sens. Actuators A Phys. 250, 258–272. 10.1016/j.sna.2016.09.028

