# Supplementary material for: Directly Printable Flexible Strain Sensors for Bending and Contact Feedback of Soft Actuators
Source: Front Robot AI. 2018 Feb 13;5:2. doi: 10.3389/frobt.2018.00002 (PMC7805767; doi:10.3389/frobt.2018.00002)
Supplement: Supplementary file 1 [file Table_1.docx]

Supplementary Material

Directly Printable Flexible Strain Sensors for Bending and Contact Feedback of Soft Actuators

Khaled Elgeneidy^1,2*^, Gerhard Neumann^2^, Michael Jackson1 and Niels Lohse^1^

^1^ EPSRC Centre for Intelligent Automation, Loughborough University, Loughborough, United Kingdom, ^2^ Lincoln Centre for Autonomous Systems, Lincoln University, Lincoln, United Kingdom

*** Correspondence:** Khaled Elgeneidy: mmkame@lboro.ac.uk

# Supplementary Tables

The following tables provide a summary for the tuned print setting that were used to successfully print air-tight bending actuators and flexible strain sensors.

| Parameter | Value |
| --- | --- |
| Layer height | 0.3 mm |
| Initial layer height | 0.3 mm |
| Initial layer line width | 100% |
| Shell thickness | 0.6 mm |
| Bottom/top thickness | 1.2 mm |
| Fill density | 100% |
| Wipe & prime tower | On, 20 mm^2^ |
| Ooze shield | Off |
| Dual extrusion switch amount | 4 mm |
| Nozzle 1 temperature (Conductive Pla) | 216^o^c |
| Nozzle 2 temperature (Ninjaflex) | 218^o^c |
| Filament 1 diameter (Conductive Pla) | 3 mm |
| Filament 2 diameter (Ninjaflex) | 3 mm |
| Bed temperature | Off |
| Support type | None |
| Platform adhesion type | None |
| Print speed | 17 mm/s |
| Retraction distance | 10 mm/s |
| Retraction speed | 4 mm |
| Z hop when retracting | 0.2 mm |

**Supplementary Table 1.** Summary of the print settings for the flexible strain sensor in dual-extrusion mode and upright orientation.

| Parameter | Value |
| --- | --- |
| Layer height | 0.3 mm |
| Initial layer height | 0.3 mm |
| Initial layer line width | 100% |
| Shell thickness | 0.6 mm |
| Bottom/top thickness | 1.2 mm |
| Fill density | 0% |
| Prime tower | Off |
| Nozzle temperature | 218^o^c |
| Filament diameter (Ninjaflex) | 3 mm |
| Bed temperature | Off |
| Support type | None |
| Print speed | 20 mm/s |
| Retraction distance | 10 mm/s |
| Retraction speed | 4 mm |

**Supplementary Table 2.** Summary of the print settings for the bending actuator in sideway orientation.
